# Isoliquiritigenin Pretreatment Induces Endoplasmic Reticulum Stress-Mediated Hormesis and Attenuates Cisplatin-Induced Oxidative Stress and Damage in LLC-PK1 Cells

**DOI:** 10.3390/molecules25194442

**Published:** 2020-09-27

**Authors:** Tania Gómez-Sierra, Omar Noel Medina-Campos, José D. Solano, María Elena Ibarra-Rubio, José Pedraza-Chaverri

**Affiliations:** Departamento de Biología, Facultad de Química, Universidad Nacional Autónoma de México, CDMX 04510, Mexico; taniags@comunidad.unam.mx (T.G.-S.); mconoel@comunidad.unam.mx (O.N.M.-C.); jdsolanobecerra@comunidad.unam.mx (J.D.S.); meir@unam.mx (M.E.I.-R.)

**Keywords:** isoliquiritigenin, cisplatin, nephrotoxicity, oxidative stress, ER stress, hormesis

## Abstract

Isoliquiritigenin (IsoLQ) is a flavonoid with antioxidant properties and inducer of endoplasmic reticulum (ER) stress. In vitro and in vivo studies show that ER stress-mediated hormesis is cytoprotective; therefore, natural antioxidants and ER stress inducers have been used to prevent renal injury. Oxidative stress and ER stress are some of the mechanisms of damage involved in cisplatin (CP)-induced nephrotoxicity. This study aims to explore whether IsoLQ pretreatment induces ER stress and produces hormesis to protect against CP-induced nephrotoxicity in Lilly Laboratories Cell-Porcine Kidney 1 (LLC-PK1) cells. During the first stage of this study, both IsoLQ protective concentration and pretreatment time against CP-induced toxicity were determined by cell viability. At the second stage, the effect of IsoLQ pretreatment on cell viability, ER stress, and oxidative stress were evaluated. IsoLQ pretreatment in CP-treated cells induces expression of glucose-related proteins 78 and 94 kDa (GRP78 and GRP94, respectively), attenuates CP-induced cell death, decreases reactive oxygen species (ROS) production, and prevents the decrease in glutathione/glutathione disulfide (GSH/GSSG) ratio, free thiols levels, and glutathione reductase (GR) activity. These data suggest that IsoLQ pretreatment has a moderately protective effect on CP-induced toxicity in LLC-PK1 cells, through ER stress-mediated hormesis, as well as by the antioxidant properties of IsoLQ.

## 1. Introduction

**Figure 1:**
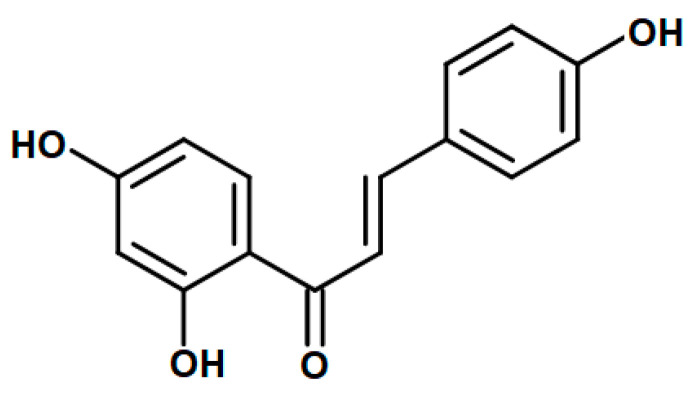
Chemical structure of isoliquiritigenin (IsoLQ).

The flavonoid 4,2′,4′-trihydroxichalcone, also known as isoliquiritigenin (IsoLQ) (Figure 1), proceeds from the roots and rhizomes of licorice (*Glycyrrizha glabra*). It is used in Asia as an herbal medicine due to its anti-inflammatory, antimicrobial, antitumoral, and antioxidant properties [1,2,3,4,5,6,7,8]. Regarding its antioxidant activity, it has been reported that IsoLQ scavenges reactive oxygen species (ROS), such as the superoxide anion (O_2_^●−^), hydroxyl radical, hydrogen peroxide, and singlet oxygen [4,7], and induces phase II cytoprotective enzymes’ expression [9,10]. In addition, IsoLQ has been used as an endoplasmic reticulum (ER) stress inducer in cancer cell lines to induce apoptosis [8,10,11,12,13,14,15,16] and also has cytoprotective effects in non-cancer cells, such as liver and kidney cells [1,4,9,16,17,18].

On the other hand, cisplatin or cis-diaminodichloroplatin II (CP), is an antineoplastic drug approved by the United States Federal Drug Administration since 1978 used for the treatment of many cancers, such as head, neck, bladder, lung, stomach, uterus, and testicular. However, its application has been limited because 25–35% of treated patients develop kidney damage [19,20,21,22,23]. The mechanism of CP nephrotoxicity has been studied for the last forty years and binding to multiple targets such as deoxyribonucleic acid or glutathione (GSH) have been identified. Also, mechanisms such as oxidative stress, inflammation, mitochondrial dysfunction, and ER stress have been associated to kidney damage [19,20,21,22,24], and although the contribution of each pathway is not exactly known, oxidative stress plays a key role [19,24,25,26].

Under acute ER stress, glucose-related protein 78 kDa (GRP78) and glucose-related protein 94 kDa (GRP94) are released from the ER membrane and activate the unfolded protein response (UPR) to restore ER functions, such as protein synthesis and transport, protein folding, lipid and steroid synthesis, among others [27,28,29,30,31], in addition to the antioxidant response and autophagy [27,32,33,34], which maintain cellular homeostasis. However, if the ER stress is excessive or chronic and cannot be corrected, UPR signaling promotes apoptosis [29,31,35,36,37,38,39]. These different effects of UPR on cellular function have generated interest because of their similarity to those occurring in hormesis, a process usually referred to as a biological favorable response originated by low dose/concentration or short exposure to chemical/natural compounds or environmental factors [40,41,42,43,44]. However, a high dose/concentration or long exposure inhibits this favorable response [40,41,45,46,47,48,49]. It has been suggested that this biphasic dose-response could protect against more than one type of stress, and play a key role in the treatment of different pathologies [36,40,41,50,51,52,53].

Based on the knowledge of the main pathways involved in CP-induced nephrotoxicity, such as oxidative stress [19,21,22,25,54], apoptosis [20,21,22,25], mitochondrial dysfunction [20,21,22,25], kinases activation [21,22,54], and ER stress [21,24,55], treatments with natural compounds have been implemented to prevent CP-induced nephrotoxicity [20,21,24,25,56,57,58]. However, it is necessary to explore more compounds and other targets less studied, such as ER stress, due to the complexity of cisplatin toxicity. Therefore, the aim of this study was to explore whether IsoLQ-induced ER stress produces a hormetic effect that protects against CP-induced nephrotoxicity in Lilly Laboratories Cell-Porcine Kidney 1 (LLC-PK1) cells.

## 2. Results

### 2.1. First Stage

#### 2.1.1. Estimation of Hormetic Zone of IsoLQ in CP-Induced Toxicity in LLC-PK1 Cells

**Figure 2:**
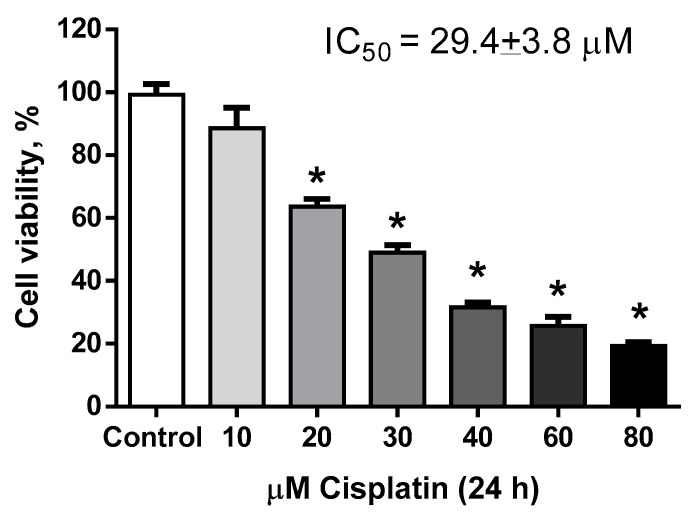
Cisplatin half-maximal (50%) inhibitory concentration (IC_50_) in 24 h treated Lilly Laboratories Cell-Porcine Kidney 1 (LLC-PK1) cells. Data are mean ± standard error of the mean (SEM), n = 3. * *p* < 0.05 vs. control (without cisplatin).

**Figure 3:**
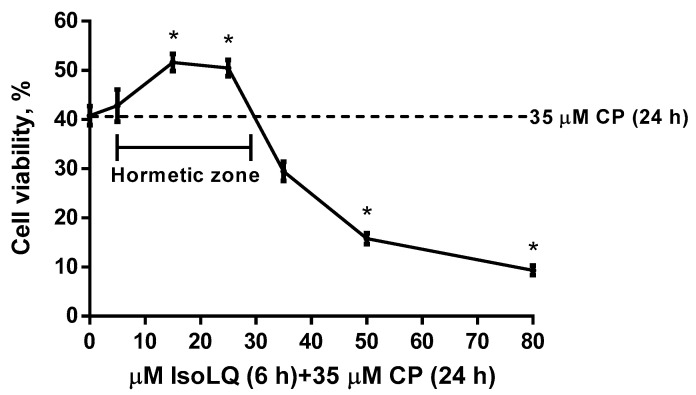
Hormetic dose-response curve of isoliquiritigenin (IsoLQ) in cisplatin (CP)-induced LLC-PK1 cell death. Data are mean ± SEM, n = 3. * *p* < 0.05 vs. CP 24 h.

LLC-PK1 cells were treated with CP (10–80 μM) for 24 h. Figure 2 shows that 10 μM CP was not cytotoxic, whereas concentrations higher than 20 μM induced cytotoxicity (*p* < 0.05) and the half-maximal (50%) inhibitory concentration (IC_50_) calculated was 29.4 ± 3.8 μM. Figure 3 presents the typical inverted J-shaped hormetic relationship, where IsoLQ concentrations between 5 and 30 μM attenuated CP-induced cell death (*p* < 0.05), whereas IsoLQ concentrations higher than 35 μM increased CP-induced cell death (*p* < 0.05).

#### 2.1.2. Determination of IsoLQ Protective Concentration and Pretreatment Time against CP-Induced Toxicity in LLC-PK1 Cells

**Figure 4:**
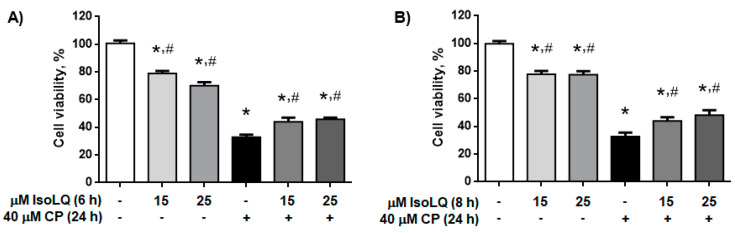
Isoliquiritigenin (IsoLQ) pretreatment attenuated cell viability decrease in cisplatin (CP)-induced toxicity. (**A**) 6 h IsoLQ pretreatment, (**B**) 8 h IsoLQ pretreatment. Data are mean ± SEM, n = 3. * *p* < 0.05 vs. control (without IsoLQ and CP), ^#^ *p* < 0.05 vs. CP.

According to the data shown in Figure 2, 40 μM CP caused ~66% of cell death (*p* < 0.05), while Figure 3 shows that 15 and 25 μM IsoLQ attenuated CP-induced decrease on cell viability (*p* < 0.05). Therefore, these two concentrations were used to determine the IsoLQ pretreatment time that could have a protective effect against CP-induced toxicity in LLC-PK1 cells. Cell viability results indicate that 6 and 8 h pretreatment with 15 and 25 μM IsoLQ promoted cytoprotective effects against 40 μM CP (*p* < 0.05) (Figure 4).

#### 2.1.3. Pretreatment of IsoLQ Induces ER Stress

**Figure 5:**
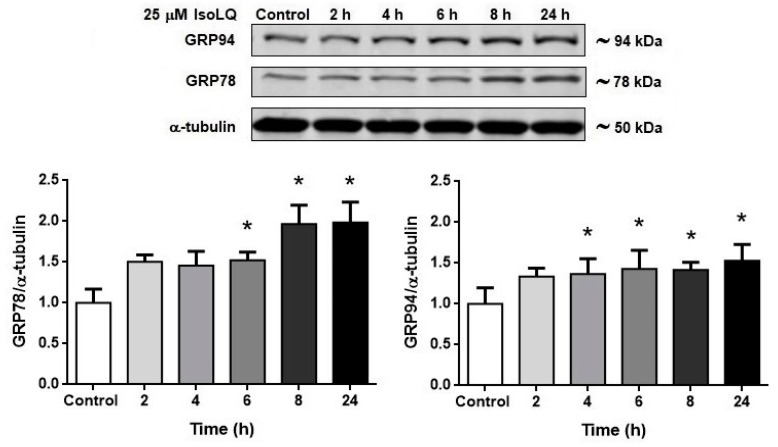
25 μM isoliquiritigenin (IsoLQ) induced endoplasmic reticulum (ER) stress in LLC-PK1 cells. Glucose-related protein 78 kDa (GRP78), glucose-related protein 94 kDa (GRP94). Data are mean ± SEM, n = 3–4. * *p* < 0.05 vs. control (without IsoLQ).

According to the data shown in Figure 4, 15 and 25 μM IsoLQ pretreatment attenuated CP-induced cell death (*p* < 0.05), so cells were treated with the highest concentration of IsoLQ (25 μM) to evaluate ER stress. Figure 5 shows that GRP94 and GRP78 increased at 4 and 6 h (*p* < 0.05) respectively, after pretreatment with 25 μM IsoLQ, and remained increased at 24 h.

### 2.2. Second Stage

#### 2.2.1. IsoLQ Pretreatment Enhanced ER Stress in CP-Induced Nephrotoxicity in LLC-PK1 Cells

**Figure 6:**
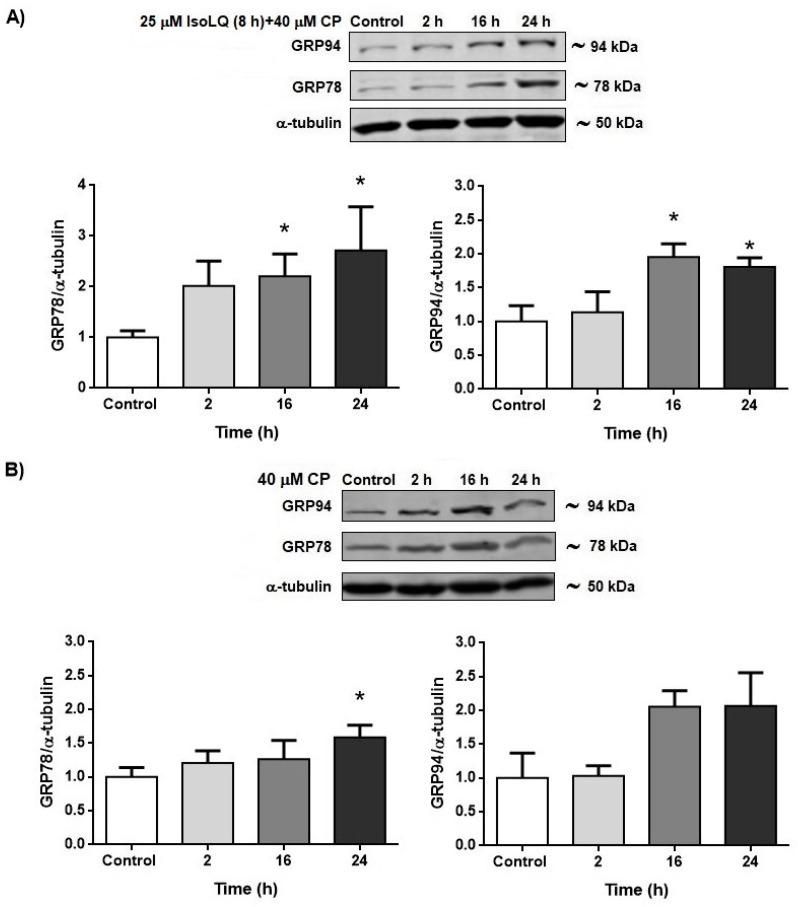
Endoplasmic reticulum (ER) stress in LLC-PK1 cells. (**A**) 25 μM isoliquiritigenin (IsoLQ, 8 h) pretreatment + 40 μM cisplatin (CP) treatment. (**B**) 40 μM CP treatment. Glucose-related protein 78 kDa (GRP78), glucose-related protein 94 kDa (GRP94). Data are mean ± SEM, n = 3. * *p* < 0.05 vs. control (without IsoLQ and CP).

Based on cell viability results as well as protein levels of GRP78 and GRP94 obtained from first-stage experiments, the experimental protocol was designed as follows: cells were pretreated with 25 μM IsoLQ for 8 h (the highest effects on cell viability and GRP78 and GRP94 expression were observed in these conditions) and subsequently with 40 μM CP (the highest CP concentration evaluated) for a time-course study. After pretreatment of LLC-PK1 cells with 25 μM IsoLQ for 8 h and exposure to 40 μM CP for 16 and 24 h, GRP78 and GRP94 expression increased (*p* < 0.05) (Figure 6A). This increase was higher than that under IsoLQ treatment or with CP alone (Figure 5 and Figure 6B, respectively). In addition, the expression of the eukaryotic initiation factor 2 *alpha* (p-eIF2α) did not change (data not shown).

#### 2.2.2. IsoLQ-Induced Hormesis Attenuates Oxidative Stress in CP-Treated LLC-PK1 Cells 

LLC-PK1 cells were pretreated with 25 μM IsoLQ for 8 h and then with 40 μM CP for 16 and 24 h, as described for the second stage of the experimental design. 25 μM IsoLQ pretreatment decreased ROS production by ~37% and ~26% after exposure to 40 μM CP for 16 and 24 h (*p* < 0.05) respectively, and attenuated the decrease in GSH/glutathione disulfide (GSSG) ratio, free thiols, and glutathione reductase (GR) activity 24 h after CP treatment (*p* < 0.05) (Table 1).

##### Nitric Oxide Synthase (NOS) is the Source of ROS Production in LLC-PK1 Cells Pretreated with IsoLQ and Exposed to CP

**Figure 7:**
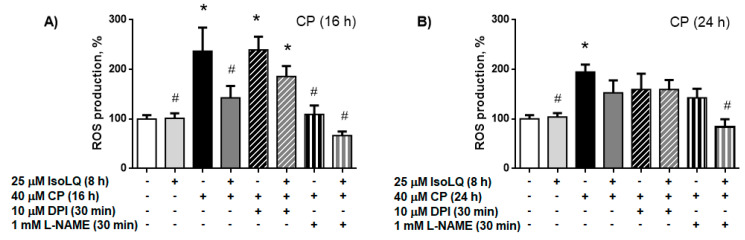
Nitric oxide synthase (NOS), but not nicotinamide adenine dinucleotide phosphate hydrogen (NADPH) oxidase (NOX), is the source of reactive oxygen species (ROS) production in LLC-PK1 cells pretreated with 25 μM isoliquiritigenin (IsoLQ, 8 h) and subsequently with 40 μM cisplatin (CP). Diphenyleneiodonium chloride (DPI), N-nitro-L-arginine methyl ester (L-NAME). (**A**) 16 h CP treatment. (**B**) 24 h CP treatment. Data are mean ± SEM, n = 3. * *p* < 0.05 vs. control (without IsoLQ and CP), ^#^ *p* < 0.05 vs. CP.

In order to determine intracellular ROS sources, we used diphenyleneiodonium chloride (DPI), an inhibitor of nicotinamide adenine dinucleotide phosphate hydrogen (NADPH) oxidase (NOX), or N-nitro-L-arginine methyl ester (L-NAME), an inhibitor of NOS. DPI did not decrease ROS production in any of the groups of treated cells, whereas L-NAME decreased ROS production at 16 h in IsoLQ + CP-treated cells to similar levels as the control group (*p* < 0.05). This effect remained until 24 h (*p* < 0.05) (Figure 7); whereas in CP-treated cells, ROS production decreased only at 16 h (*p* < 0.05). 

## 3. Discussion

IsoLQ is a flavonoid with anti-inflammatory, antitumoral, and antioxidant properties [1,2,3,4,5,6,7,16,59] that exerts protective effects in liver and kidney cells [3,4,9,16,17,18]. In addition, it has been demonstrated that IsoLQ does not interfere with CP antineoplastic activity [16]. The effect of IsoLQ on cell viability in CP-induced nephrotoxicity was a biphasic dose-response: low IsoLQ concentrations attenuated the decrease on cell viability, but high IsoLQ concentrations inhibited it. This effect is known as hormesis, a term that refers to a biological favorable cellular response [40,47,48,49,50,60]. The IsoLQ + CP dose-response curve showed an inverted J-shaped hormetic effect and this biphasic curve has an interval called the hormetic zone where the beneficial effects are evident (Figure 3) [45,47,48,49]. The low-dose stimulation reflects the capacity of the cells to distribute biological resources that help to defend the organism from a wide range of stressor agents, such as CP, and this adaptive mechanism includes stress protein responses that reduce the damage more effectively than the response induced only by exposure to the stressor [40,41,46,47]. Therefore, hormesis is more than simply a dose-response relationship, it is a quantitative and temporal manifestation of reparative processes that is adaptive in nature, such as UPR elicited by ER stress [42,43,46,47,50,60,61].

Data obtained show that 25 μM IsoLQ pretreatment induced ER stress and also attenuated 40 μM CP-induced cell death in LLC-PK1 cells. The increase in the expression of the chaperones GRP78 and GRP94 indicates the occurrence of ER stress and suggests the activation of UPR [27,29,37,62,63,64,65]. The early induction of ER stress could play a key role in cell survival, because ER stress induced by IsoLQ pretreatment generated a hormetic effect to activate adaptive responses such as UPR, that protects against higher stress levels as occurs in CP-induced nephrotoxicity. In in vitro and in vivo models of kidney diseases, like renal ischemia-reperfusion or exposure to cytotoxic compounds, the a priori induction of ER stress with pharmacological or natural compounds generates tolerance against these insults (hypoxia, oxidative stress) in order to inhibit the apoptotic cell death [37,52,53,62,66,67,68,69].

On the other hand, oxidative stress is also characteristic in CP-induced nephrotoxicity and is an important factor in the development and progression of other associated damage mechanisms, such as mitochondrial dysfunction, inflammation, and ER stress [19,20,21,22,25,26,54,70]. ROS are generated naturally and regulate cell functions [71,72]. However, excessive quantities of ROS can result in damage to proteins, modifying their function and disrupting ER homeostasis [27,32,34,73]. ROS overproduction can be sensed by the ER, which is highly sensitive to changes in ROS levels through redox sensors, such as, for example, the thiol groups of cysteines [33,70,74,75,76,77], causing misfolded protein accumulation and consequently increasing ROS production, which leads to a vicious cycle able to activate UPR, generate chronic stress, and induce apoptosis [32,33,34,70,72]. Moreover, ROS overproduction causes a decrease in the GSH/GSSG ratio and in antioxidant enzyme levels, whilst oxidizing lipids, DNA, and proteins [19,20,21,22,25,26]. In our experimental model, ROS production could be associated to NOS activity and consequently to overproduction of O_2_^●−^ that enhances oxidative stress. However, IsoLQ can attenuate this oxidative imbalance due to its ROS scavenging capacity [4,7]. In CP-induced nephrotoxicity, the decrease in the GSH/GSSG ratio is characteristic due to the formation of GSH-CP adducts. The positively charged species of CP (their active forms) have a great affinity for thiol groups [20,22,25,78,79] and consequently, there is an alteration in the activity of enzymes related to GSH metabolism [19,26,78]. In addition, the decrease in GSH could be associated with the formation of disulfide bonds among misfolded proteins in a coupled reaction with the protein disulfide isomerase and ER oxidoreductase 1 [73,80,81,82,83].

**Figure 8:**
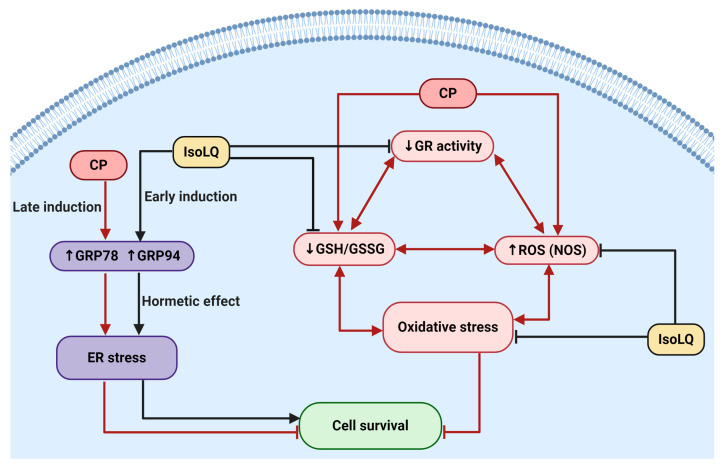
Mechanisms involved in the protective effect of isoliquiritigenin (IsoLQ) against cisplatin (CP)-induced damage in LLC-PK1 cells. Pretreatment with IsoLQ increased the expression of glucose-regulated protein 78 (GRP78) and glucose-regulated protein 94 (GRP94), markers of endoplasmic reticulum (ER)-stress. The generated hormetic effect induces a decrease in reactive oxygen species (ROS) production, associated to nitric oxide synthase (NOS) activity, as well as the attenuation in the reduction of glutathione/glutathione disulfide (GSH/GSSG) ratio and glutathione reductase (GR) activity. These combined effects, in addition to the antioxidant properties of IsoLQ, attenuated oxidative stress. Figure was created with Biorender.com.

Interestingly, ER stress induction is also associated with an increase in GSH biosynthesis in order to form disulfide bonds between proteins and recycle unfolded proteins. This effect could explain the increase in free thiols, such as GSH, in cells pretreated with IsoLQ [33,82]. Moreover, it has been described that ER stress increases the activity of antioxidant enzymes such as GR, glutathione peroxidase, and glutathione S-transferase [33,63,82]. Probably, augmented GR activity in cells pretreated with IsoLQ was due to this effect, since IsoLQ is also a bifunctional antioxidant able to induce expression of phase II detoxifying enzymes through nuclear factor erythroid 2 (Nrf2) transcriptional regulation [9,10]. Under ER stress, protein kinase ribonucleic acid (RNA)-activated-like ER kinase (PERK), one the of the signaling pathways of UPR, directly phosphorylates Nrf2, causing Nrf2 to dissociates from its negative regulator (Kelch-like erythroid-derived cap-n-collar homology-(ECH-)associated protein 1); after which, Nrf2 translocates to the nucleus, leading the transcription of genes that encode phase II detoxifying enzymes that maintain redox homeostasis and may contribute to cell survival [84,85,86]. In addition, eIF2α is another target of the PERK signaling pathway [27,29,63,65]. Even though the data on p-eIF2α expression are inconclusive (data not shown), it is possible that the PERK pathway has become activated. Nevertheless, studies on the role of UPR proteins are required to describe and understand the mechanisms involved in the hormetic effect produced by IsoLQ in this experimental model. In summary, Figure 8 shows a possible mechanism of the protective effect of IsoLQ against CP-induced nephrotoxicity in LLC-PK1 cells. We derived our assumptions from the main observations found in this study.

## 4. Materials and Methods

### 4.1. Reagents

LLC-PK1 cells were obtained from the American Type Culture Collection (ATCC, Rockville, MD, USA). IsoLQ was purchased from AK Scientific (Union City, NJ, USA). Bovine serum albumin, CP, 1-chloro-2,4-dinitrobenzene, dimethyl sulfoxide, DPI, 5,5’-dithiobis-(2-nitrobenzoic acid), fluorescein diacetate (FDA), glycerophosphate, GSH, GSSG, GR, L-NAME, NADPH, 4-nonylphenyl-polyethylene glycol (NP-40), protease inhibitor cocktail, sodium deoxycholate, sodium dodecyl sulfate (SDS), sodium fluoride (NaF), sodium pyrophosphate (Na_4_P_2_O_7_), sodium orthovanadate (Na_3_VO_4_), sulfosalicylic acid, triethanolamine, Tris-hydrochloride (Tris-HCl), trypan blue, triton X-100, tween 20, 2-vinylpirydine, mouse anti-α-tubulin (cat. T9026) antibodies, and rabbit anti-GRP94 (cat. G4420) antibodies were from Sigma Aldrich Co. (St. Louis, MO, USA). Dipotassium hydrogen orthophosphate, ethylenediaminetetraacetic acid disodium salt (EDTA), potassium chloride, potassium dihydrogen orthophosphate, sodium chloride (NaCl), sodium bicarbonate (NaHCO_3_), and sodium phosphate dibasic were from J.T. Baker (Xalostoc, Edo. Mex, Mex). Bradford reagent was from Bio-Rad (Hercules, CA, USA). Dulbecco’s Modified Eagle’s Medium (DMEM), fetal bovine serum (FBS), and penicillin/streptomycin were from Biowest (Riverside, MO, USA). TrypLE Express was from Thermo Fisher Scientific (Waltham, MA, USA). Dichlorodihydrofluorescein diacetate (H_2_DCFDA) was from Molecular Probes (Eugene, OR, USA). Mouse anti-GRP78 (cat. SC-3768) antibodies were from Santa Cruz Biotechnology Inc. (Dallas, TX, USA). Fluorescent secondary antibodies anti-rabbit 800CW and anti-mouse 680RD were from LI-COR Biosciences (Lincoln, NE, USA). All the other chemicals and reagents used were of analytical grade and commercially available.

### 4.2. Cell Culture

LLC-PK1 cells are a proximal tubule cell line derived from porcine kidneys, widely used in experimental models [9,25,53,87]. LLC-PK1 cells were cultured in DMEM supplemented with 10% FBS, 0.33% NaHCO_3_, 100 U/mL penicillin, and 50 U/mL streptomycin under a humidified atmosphere of 5% CO_2_ at 37 °C. The cells were sub-cultured with TrypLE Express upon reaching 90% confluence. LLC-PK1 cells passage numbers 8 to 33 were used.

### 4.3. Experimental Design 

#### 4.3.1. First Stage

The aim of the first stage set of experiments was to determine the potential IsoLQ protective concentration and pretreatment time against CP-induced toxicity in LLC-PK1 cell culture. Cells were treated with CP (10–80 μM) for 24 h to calculate the IC_50_. Additionally, cells were pretreated with IsoLQ (5–80 μM) for 6 h and later incubated with one concentration higher than the IC_50_ of CP (35 μM) for 24 h to evaluate the potentially hormetic dose-response curve for cell viability. Finally, two IsoLQ concentrations within the hormetic zone (15 and 25 μM) and one concentration higher than the IC_50_ of CP (40 μM) were selected to test the putative protective effect by IsoLQ on cell viability.

On the other hand, cells were pretreated with 25 μM IsoLQ (highest concentration within the hormetic zone) in a time-course study (2–24 h) to evaluate the expression of GRP78 and GRP94.

#### 4.3.2. Second Stage

**Figure 9:**
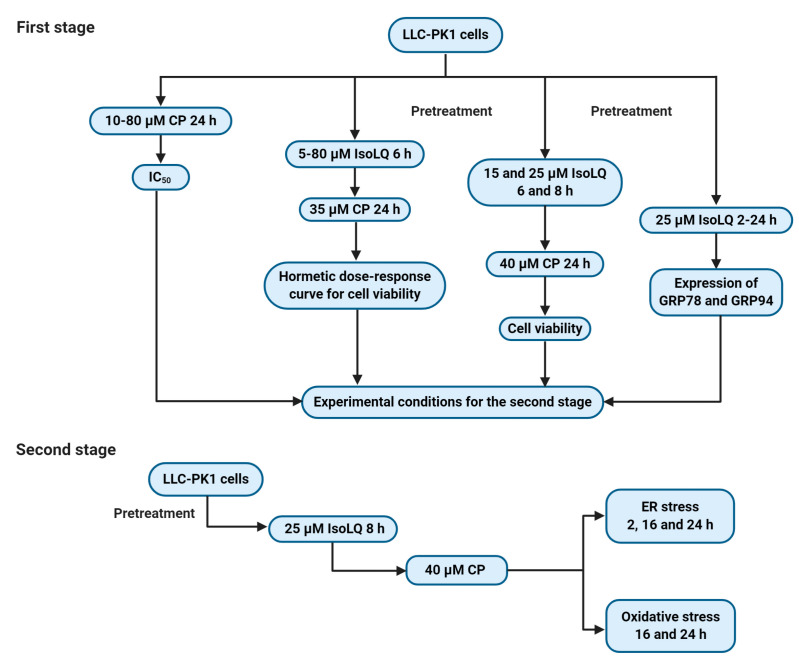
Scheme of the experimental design. CP, cisplatin; IsoLQ, isoliquiritigenin; GRP78, glucose-regulated protein 78 kDa; GRP94, glucose-regulated protein 94 kDa; ER, endoplasmic reticulum. Figure was created with Biorender.com.

Based on the first stage results, LLC-PK1 cells were pretreated with 25 μM IsoLQ for 8 h and subsequently incubated, in a time-course study, with 40 μM CP to evaluate ER stress (2, 16, and 24 h) and oxidative stress (16 and 24 h). The experimental design is summarized in Figure 9.

### 4.4. Cell Viability

Subsequently after treatments, DMEM was removed and cells were washed with phosphate-buffered saline (PBS) followed by the addition of 12 μM FDA and incubation for 5 min at 37 °C in darkness [88]. Fluorescence was determined by a microplate reader at excitation and emission wavelengths of 485 and 520 nm, respectively. The results are expressed as the percentage of the control (without treatments).

### 4.5. Extraction of Total Fractions of Proteins for Western Blot

LLC-PK1 cells were lysed with radioimmunoprecipitation assay (RIPA) buffer (50 mM Tris-HCl, pH 7.4, 150 mM NaCl, 1 mM EDTA, 0.5% sodium deoxycholate, 1% NP-40, 0.1% SDS, 25 mM NaF, 1 mM Na_4_P_2_O_7_, 1 mM Na_3_VO_4_, 0.5 mM glycerophosphate, and protease inhibitor cocktail) for one hour at 4 °C. Lysates were centrifuged at 12,000× *g* during 10 min at 4 °C and the supernatant was collected and stored at −70 °C until use. Total protein content was determined by the Bradford assay [89].

### 4.6. ER Stress Evaluation

#### Western Blot Analysis

Proteins were separated by SDS-polyacrylamide gel electrophoresis (PAGE) and transferred to polyvinylidene fluoride membranes, which were blocked for one hour with 5% skimmed milk or bovine serum albumin. Membranes were left incubating overnight at 4 °C with the appropriate primary antibodies against GRP78 (1:1000), GRP94 (1:1000), and α-tubulin (1:8000). Subsequently, membranes were washed and incubated with the appropriate secondary antibodies for two hours. The proteins of interest were detected with the LI-COR Odyssey Sa Infrared Imaging System (Lincoln, NE, USA). Image analysis was performed with Image Studio Lite Software version 5.2.

### 4.7. Oxidative Stress Evaluation

#### 4.7.1. Measurement of ROS Production 

After the second stage cell treatments, DMEM was removed and cells were washed with PBS followed by incubation with 10 µM H_2_DCFDA for 30 min at 37 °C in the darkness. Fluorescence intensity was visualized and determined in a Cytation 5 Cell Imaging Reader (Biotek Instruments, Inc., Winooski, VT, USA) at excitation and emission wavelengths of 485 and 520 nm, respectively. Relative fluorescence intensity of treated cells was expressed as a percentage of the control (without treatments).

In order to determine the intracellular ROS sources, LLC-PK1 cells were pretreated with IsoLQ for 8 h, then with CP for 16 or 24 h. At 30 min before the end of the CP treatments, the NOX inhibitor 10 μM DPI and the NOS inhibitor 1 mM L-NAME, were added separately to the cell cultures. Finally, the growth medium was removed, and the measurement of ROS was carried out as described above.

#### 4.7.2. Measurement of GSH/GSSG Ratio

The redox status, expressed as the GSH/GSSG ratio, was determined following a method previously described [90].

#### 4.7.3. Free Thiols Levels

Free thiols were determined following a method previously described [91]. Total protein content was determined by the Bradford assay [89]. Results are expressed as nmol/mg protein.

#### 4.7.4. GR Activity

Activity of GR was determined by measuring the disappearance of NADPH at 340 nm. Total protein content was determined by the Bradford assay [89]. Results are expressed as U/mg protein, where U equals one μmol of oxidized NADPH per minute [92].

### 4.8. Statistical Analysis

The results are expressed as mean ± standard error of the mean (SEM). Statistical analysis was performed for each determination using the software Graph-Pad Prism 6.0 (San Diego, CA, USA) comparing the mean values through a one-way analysis of variance, followed by the Tukey’s multiple comparison test. For the time-course studies of Western blot, a Student’s *t*-test was carried out with respect to the control. A *p*-value < 0.05 was considered significant.

## 5. Conclusions

The mechanism of cisplatin toxicity is complex and involves many cellular targets, some of which are less studied, such as ER stress, which could have cytoprotective or cytotoxic effects. However, if ER stress is induced early with natural compounds such as IsoLQ, the effects could be beneficial because the cell adapts to stress, distributing biological resources to help defend the organism from a wide range of stressor agents, such as CP. However, to describe the mechanisms involved in this specific hormetic effect, it is necessary to study and explore each UPR pathway activated in this particular experimental model.

In conclusion, our results suggest that pretreatment with IsoLQ induces the expression of GRP78 and GRP94, consequently inducing ER stress-mediated hormesis and ameliorating oxidative stress, both of which have a moderately protective effect against CP-induced nephrotoxicity in LLC-PK1 cells. This study provides another strategy or perspective to prevent cisplatin-induced toxicity, which can be applied to the study of other reticulum stress-inducing compounds.

## Figures and Tables

**Table 1:** Effect of isoliquiritigenin (IsoLQ)-induced hormesis on oxidative stress in LLC-PK1 cells after 16 and 24 h of cisplatin (CP) treatment.

Groups	Control	25 μM IsoLQ (8 h)	40 μM CP		25 μM IsoLQ (8 h) + 40 μΜ CP	
Time CP Treatment	0 h	0 h	16 h	24 h	16 h	24 h
ROS production, %	100 ± 4.5	100.1 ± 6.7 **^,#^	236.5 ± 36.2 *	206.8 ± 24.5 *	147.9 ± 18.2 *^,^**	152.3 ± 24.5 *
GSH/GSSG	5.92 ± 0.35	ND	1.67 ± 0.19 *	1.49 ± 0.16 *	2.14 ± 0.13 *	3.22 ± 0.44 *^,#^
Free thiols (nmol/mg protein)	143.9 ± 2.9	138.1 ± 13.6 ^#^	115.1 ± 3.6	85.1 ± 4.6 *	148.9 ± 27.1	204.9 ± 28.7 ^#^
GR activity (U/mg protein)	0.015 ± 0.001	0.014 ± 0.001 ^#^	0.010 ± 0.001 *	0.006 ± 0.001 *	0.011 ± 0.001 *	0.014 ± 0.001 ^#^

ROS, reactive oxygen species; GSH/GSSG, glutathione/glutathione disulfide ratio; GR, glutathione reductase. Data are mean ± SEM, n = 3. * *p* < 0.05 vs. control (without IsoLQ and CP), ** *p* < 0.05 vs. CP 16 h, ^#^ *p* < 0.05 vs. CP 24 h. ND, not determined.
